# Incomplete Ionization of a 110 meV Unintentional Donor in β-Ga_2_O_3_ and its Effect on Power Devices

**DOI:** 10.1038/s41598-017-13656-x

**Published:** 2017-10-16

**Authors:** Adam T. Neal, Shin Mou, Roberto Lopez, Jian V. Li, Darren B. Thomson, Kelson D. Chabak, Gregg H. Jessen

**Affiliations:** 1 Air Force Research Laboratory, Materials and Manufacturing Directorate, Wright Patterson AFB, OH USA; 2 Universal Technology Corporation, Dayton, OH USA; 3 Texas State University, Department of Physics, San Marco, TX USA; 4 Air Force Research Laboratory, Sensors Directorate, Wright Patterson AFB, OH USA

## Abstract

Understanding the origin of unintentional doping in Ga_2_O_3_ is key to increasing breakdown voltages of Ga_2_O_3_ based power devices. Therefore, transport and capacitance spectroscopy studies have been performed to better understand the origin of unintentional doping in Ga_2_O_3_. Previously unobserved unintentional donors in commercially available $(\bar{2}01)$ Ga_2_O_3_ substrates have been electrically characterized via temperature dependent Hall effect measurements up to 1000 K and found to have a donor energy of 110 meV. The existence of the unintentional donor is confirmed by temperature dependent admittance spectroscopy, with an activation energy of 131 meV determined via that technique, in agreement with Hall effect measurements. With the concentration of this donor determined to be in the mid to high 10^16^ cm^−3^ range, elimination of this donor from the drift layer of Ga_2_O_3_ power electronics devices will be key to pushing the limits of device performance. Indeed, analytical assessment of the specific on-resistance (R_onsp_) and breakdown voltage of Schottky diodes containing the 110 meV donor indicates that incomplete ionization increases R_onsp_ and decreases breakdown voltage as compared to Ga_2_O_3_ Schottky diodes containing only the shallow donor. The reduced performance due to incomplete ionization occurs in addition to the usual tradeoff between R_onsp_ and breakdown voltage.

## Introduction

While crystalline Ga_2_O_3_ has been known for many years, the recent availability of high quality crystalline substrates^1^ and the demonstration of Ga_2_O_3_ MESFETs^2^ and MOSFETs^3–6^ have motivated interest in Ga_2_O_3_ for next generation ultra-wide bandgap power electronics applications. With a bandgap of 4.5–4.9 eV and estimated critical breakdown field of 8MV/cm, Ga_2_O_3_ possesses a Baliga figure of merit 10 times greater than SiC and four times greater than GaN^2^. Indeed, even at this early stage of development, electric fields of at least 3.8 MV/cm^4^ and 5.1 MV/cm^7^ have been demonstrated in lateral Ga_2_O_3_ FETs and vertical Schottky diodes, respectively, already surpassing bulk critical fields of GaN and SiC. In addition to these promising material properties, melt-growth methods for bulk Ga_2_O_3_ substrate growth^8–17^ are expected to be more cost-effective than the sublimation techniques used for the growth of SiC substrates, lowering manufacturing costs for Ga_2_O_3_ based power electronics.

**Figure 1:**
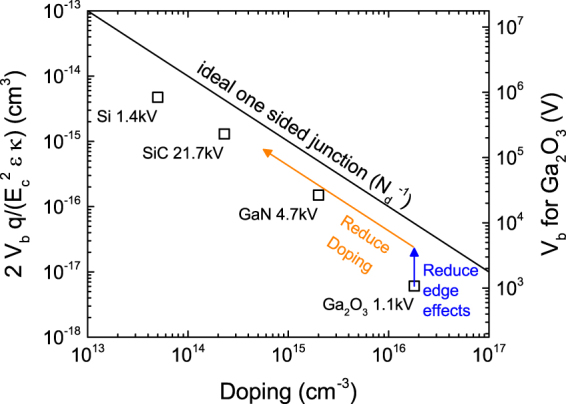
Normalized breakdown voltage (left axis) vs. doping concentration for single-sided junction power devices. The ideal relationship assuming the full-depletion approximation and 1D electrostatics is shown by the black line. The right axis indicates the projected absolute breakdown voltage for Ga_2_O_3_ devices for comparison. Square symbols indicate experimental measurements of devices with breakdown voltages among the highest reported for each material: Si^38^, SiC^39^, GaN^40^, Ga_2_O_3_^7^.

While the large breakdown electric field demonstrated in these early experimental studies is certainly promising, pushing the breakdown voltages of Ga_2_O_3_ based power electronics devices towards their predicted limits requires further work to understand and eliminate unintentional doping in the material. To illustrate this fact, the dependence of breakdown voltage of p-n single-sided junction and Schottky junction devices, normalized by material parameters, is plotted in Fig. 1. The black line indicates the theoretical limit, assuming the full-depletion approximation and one dimensional electrostatics. By normalizing the breakdown voltages in this way, one can isolate the effect of doping on device breakdown voltage to allow comparisons of devices made from different materials. Comparing early Ga_2_O_3_ devices to those of more mature semiconductor materials gives insight into the future potential of Ga_2_O_3_ devices. The plotted experimental data are among the best reported for vertical devices of each material with large breakdown voltage and low doping of the device drift-layer. While edge effects prevent real devices of any material from matching the simplified theoretical limit exactly, the ${{N}_{d}}^{-1}$ trend is nevertheless observed considering the best performing devices of the different material systems. Comparing Ga_2_O_3_ to the more mature materials, it is clear that some improvement can be expected from the optimization of device geometries to mitigate edge effects, as shown by the blue arrow. However, much greater improvement in breakdown voltage is possible from reducing drift layer doping in Ga_2_O_3_ devices, as indicated by the orange arrow. For instance, if the drift layer doping of a Ga_2_O_3_ device can be reduced to about 10^14^ cm^−3^ like the 21.7 kV SiC device indicated in Fig. 1, then the Ga_2_O_3_ device will have a breakdown voltage over 100 kV as indicated by the right axis of Fig. 1. Therefore, understanding and mitigating unintentional doping in Ga_2_O_3_ is key to increasing the maximum achievable breakdown voltages in Ga_2_O_3_ beyond the recently demonstrated 1 kV devices^7^.

With these motivations, we have undertaken transport and capacitance spectroscopy studies in order to better understand the origin of unintentional doping in Ga_2_O_3_. The transport properties of commercially available unintentionally doped $(\bar{2}01)$ Ga_2_O_3_ substrates from Tamura Corporation grown via the EFG method^13,14^ are characterized via temperature dependent Hall effect measurements. High temperature Hall effect measurements up to 1000 K reveal a previously unobserved unintentional donor with an energy level 110 meV below the conduction band edge in addition to previously observed shallow donors attributed to Si^14,17^. To confirm the existence of this unintentional donor, temperature dependent admittance spectroscopy measurements were performed on a Ga_2_O_3_ Schottky diode structure, with an activation energy of 131 meV observed via this technique. This activation energy agrees well with the donor energy of 110 meV determined via Hall effect measurements. Finally, using the information obtained from the temperature dependent Hall effect measurements, the effects of the 110 meV donor on the specific on-resistance (R_onsp_) of Ga_2_O_3_ Schottky diodes are assessed via analytical calculations, indicating that incomplete ionization of the 110 meV donor increases the R_onsp_ and reduces breakdown voltage as compared to Ga_2_O_3_ devices with only the shallow donor, beyond the usual tradeoff between on-resistance and breakdown voltage.

## Results

### Hall Effect Measurements

**Figure 2:**
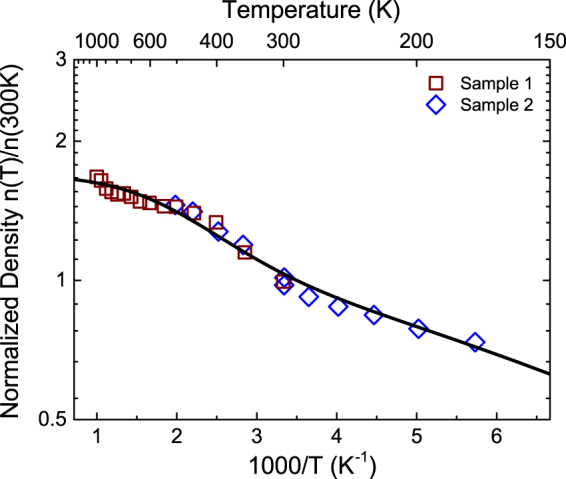
Hall carrier density (log scale) vs. 1000/T of Ga_2_O_3_ for two samples measured in the square geometry. The data are normalized to the Hall carrier density at 300 K. The symbols are the measured data and the black line a fit. Room temperature electron densities are 1.71 × 10^17^ cm^−3^ for Sample 1 (red square) and 1.21 × 10^17^ cm^−3^ for Sample 2 (blue diamond).

Figure 2 shows the temperature dependent carrier density, normalized to room temperature, as measured by the Hall effect for two samples. The Hall scattering factor was assumed to be one when calculating the carrier density. The temperature dependent conductivity, mobility, and analysis of the relevant scattering mechanisms for these same samples can be found in the supplementary information. With the increased slope of the log-reciprocal plot from room temperature up to about 450 K, the temperature dependence of the carrier density must be determined by two donors with different energy levels. To estimate the donor energy levels, the data was fit with a model consisting of two donors with a compensating acceptor as shown in Equation (1):

1 $${N}_{c}{e}^{\frac{{E}_{f}-{E}_{c}}{kT}}+{N}_{a}=\frac{{N}_{d1}}{1+2{e}^{\frac{{E}_{f}-{E}_{d1}}{kT}}}+\frac{{N}_{d2}}{1+2{e}^{\frac{{E}_{f}-{E}_{d2}}{kT}}}$$

where ${N}_{c}$ is the effective density of states in the conduction band, ${N}_{d1}\,$and ${N}_{d2}$ the concentrations of the two donors, ${N}_{a}$ the concentration of compensating acceptors, ${E}_{c}$ the energy of the conduction band edge,$\,{E}_{f}$ the Fermi level, ${E}_{d1}$ and ${E}_{d2}$ the donor energies. An effective mass *m* ^*^ = 0.3*m*_0_^18–20^ was used to estimate ${N}_{c}$ analytically^21^, while ${N}_{d1}$, ${N}_{d2}$, ${E}_{d1}$, ${E}_{d2}$, and ${N}_{a}$ are free parameters. The values of the free parameters are summarized in Table 1, and the resulting fit is plotted as a black line in Fig. 2. The model indicates that the increased slope from 300 K to 450 K is the result of a higher energy donor with energy 110 meV. Additionally, a shallow donor is also observed with energy 23 meV, previously identified as silicon from glow discharge mass spectrometry (GDMS) analysis of Tamura samples^22^. Carrier activation in previous studies of Ga_2_O_3_ was well described by a single shallow donor which they also attributed to unintentional silicon dopants;^14,17^ however, the second higher energy donor has not been observed previously.

**Table 1:** Parameters for the carrier density vs. temperature model.

N_*d*1_	(cm^−3^)	7.5 × 10^16^
E_*c*_−*E*_*d*1_	(meV)	110
N_*d*2_	(cm^−3^)	1.4 × 10^17^
E_*c*_−*E*_*d*2_	(meV)	23
N_*a*_	(cm^−3^)	1.0 × 10^16^

### Admittance Spectroscopy Measurements

**Figure 3:**
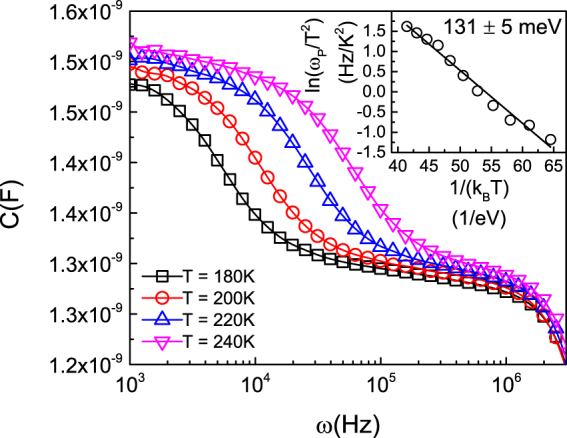
Frequency dependent capacitance (C-f) data at different temperatures (T) indicating the unintentional donor. Inset: Arrhenius plot [ln(*ω*_*p*_/T^2^) vs 1/(k_B_T)] for the trap signature observed through admittance spectroscopy in which *ω*_p_ is the negative peak in the *ωdC/dω* vs *ω* spectrum. The activation energy is extracted from the slope (fitted line). This activation energy matches that determined for the unintentional donor via Hall effect measurement.

Admittance spectroscopy measurements confirm the existence of the previously unobserved donor. Figure 3 shows capacitance of a Ga_2_O_3_ Schottky diode as a function of frequency for several temperatures. The *C* vs. *ω* spectrum exhibits a step transition separating two plateaus, *C*_*d*_ at *ω* < *ω*_*p*_ and *C*_*g*_ at *ω* > *ω*_*p*_, which indicates the presence of a trap state. The transition frequency, *ω*_*p*_, corresponds to the carrier emission rate from the trap level. To determine the activation energy of the trap state, *ω*_*p*_ was obtained from the negative peak in the differential capacitance (*ωdC/dω* vs *ω*) spectrum to construct an Arrhenius plot of ln(*ω*_*p*_/T^2^) vs 1/(k_B_T), shown in Fig. 3 inset. From the slope of the plot, the activation energy of the trap is determined to be *E*_*a*_ = 131 ± 5 meV. Furthermore, the trap density, calculated from the height of the capacitance step^23^, is *N*_*t*_ = 4.4 × 10^16^ cm^−3^. A relative dielectric constant of 10 for Ga_2_O_3_ was used for this calculation^24,25^. These data are well matched to the previously unobserved donor identified via Hall effect measurements, which indicated a donor energy of 110 meV and donor density of 7.5 × 10^16^ cm^−3^ as previously discussed. The small differences between the Hall effect measurements and admittance spectroscopy measurements are not surprising and are consistent with sample to sample variation within the two-inch Ga_2_O_3_ wafer from which the three samples were cut. Therefore, the trap state observed via admittance spectroscopy confirms the existence of the previously unobserved unintentional donor.

### Possible Origins of the Donor

While the electrical characterization performed here cannot determine the chemical or crystallographic origin of the 110 meV donor, we nevertheless consider a few hypotheses. It is natural to ask if a native defect is responsible for the observed donor. DFT calculations rule out simple oxygen and gallium vacancies as they are determined to be deep donors^26^ and acceptors^27,28^, respectively. However, antisites and interstitials could be responsible. Extrinsic impurities, of course, could also be responsible for the donor, and glow discharge mass spectrometry (GDMS) analysis of Tamura substrates indicates the presence of several impurities whose dopant properties in Ga_2_O_3_ are unknown^22^. Last, silicon on the octahedrally coordinated Ga(II) site of Ga_2_O_3_ could be responsible for the 110 meV donor. DFT calculations indicate that Si prefers the tetrahedrally coordinated Ga(I) site in which it is a shallow donor;^26^ however, STM analysis of Si donors near the (100) surface of Ga_2_O_3_ indicates that Si occupies both Ga(I) and Ga(II) lattice sites^29^. This fact suggests that Si on the Ga(II) site should also be considered as a possible origin of the 110 meV donor.

## Discussion

These unintentional donors have implications for the performance of Ga_2_O_3_ based power devices; namely they reduce the maximum achievable breakdown voltage in a Ga_2_O_3_ power device. Because the doping of the drift-layer cannot be reduced below the level of unintentional doping, the density of unintentional donors sets the lower limit on electric field at the metal-semiconductor interface and the upper limit on the depletion width of the Ga_2_O_3_ drift-layer for a given applied voltage. Therefore, a limit is set on the tradeoff which allows higher breakdown voltage to be achieved at the expense of larger on-resistance. To mitigate the presence of previously identified silicon shallow donors in the substrate, several methods including LPCVD^30^, MOCVD^31,32^, HVPE^33,34^, and MBE^2,3,35,36^ have been used to grow homoepitaxial Ga_2_O_3_ layers on Ga_2_O_3_ substrates, achieving lower carrier densities by minimizing unintentional doping. However, the carrier concentration due to unintentional doping still remaining in epitaxial layers is typically 10^15^ to 10^17^ cm^−3^ ^37^. This concentration is similar to the additional carrier concentration due to the 110 meV donor observed in these bulk samples considering incomplete ionization. For example, at a 110 meV donor concentration of 7.5 × 10^16^ cm^−3^ as found in this study, with no other donors or acceptors, 3.4 × 10^16^ cm^−3^ free electrons are thermally activated into the conduction band at 300 K for an ionization efficiency of 46%. These facts suggest that the 110 meV donor may also play a role in the unintentional doping of epitaxially grown Ga_2_O_3_, warranting further study but beyond the scope of this work. The importance of the high temperature Hall effect measurements and admittance spectroscopy measurements should be emphasized. As our calculation indicates, the higher activation energy of the 110 meV donors means that their ionization efficiency ${N}_{d}^{+}/{N}_{d}$ is significantly less than 100% at room temperature. Therefore, room temperature and low temperature Hall measurement will not identify the full donor concentration, ${N}_{d}$, which determines the maximum breakdown voltage. Underestimating the concentration of donors could lead to an overestimate of the breakdown voltage without high temperature Hall effect measurements or admittance spectroscopy measurements like those performed in this study.

**Figure 4:**
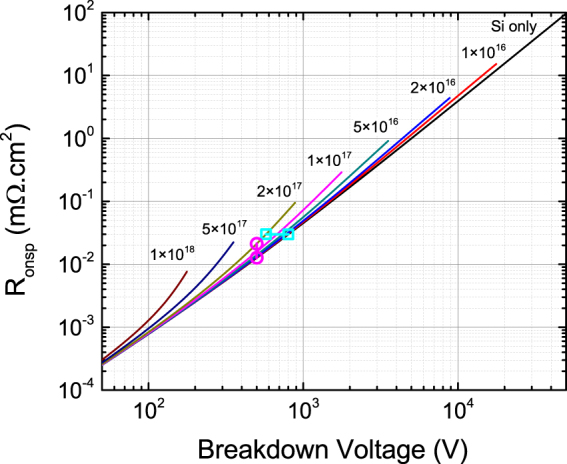
Analytical calculation of specific on-resistance (R_onsp_) vs. breakdown voltage for Ga_2_O_3_ based Schottky junction devices with 110 meV donors including the effects of incomplete ionization. The label for each curve indicates the fixed concentration of 110 meV donors in units of cm^−3^. The pink circles, cyan squares, and line segments illustrate the relationship to Fig. 5 and Figure S3 as described in the supplementary information.

**Figure 5:**
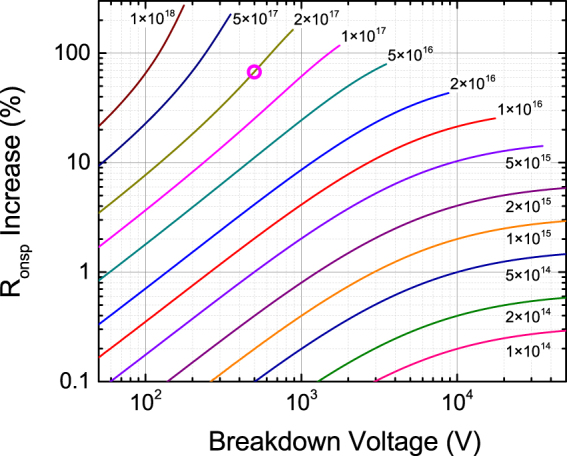
Percent increase in specific on-resistance (R_onsp_) due to incomplete ionization as a function of breakdown voltage comparing Ga_2_O_3_ Schottky diodes with 110 meV donors to those without. The labels indicate the fixed concentration of 110 meV donors in cm^−3^ for each curve. The pink circle illustrates the relationship to Fig. 4 as described in the supplementary information.

While unintentional shallow donors and the 110 meV donor both set a limit on the tradeoff between on-resistance and breakdown voltage, the 110 meV donor poses an additional challenge to maximizing breakdown voltage and minimizing on-resistance due to its four times larger donor energy as compared to the shallow donor. Because of the higher donor energy, incomplete ionization of the 110 meV donor is more severe than for the shallow donor, degrading the specific on-resistance vs. breakdown voltage characteristic for Ga_2_O_3_ based devices. The 110 meV donor is shallow enough in energy that it becomes fully ionized when the Schottky diode is reverse biased, reducing the depletion width, increasing the electric field at the metal-semiconductor interface, and reducing the breakdown voltage in the same way as the shallow donor. However, the 110 meV donor energy is large enough that incomplete ionization can be significant, meaning that only a fraction of the 110 meV donors are ionized when the Schottky diode is forward biased, reducing the carrier concentration available to conduct the on-current. To quantitatively examine those effects, specific on-resistance (R_onsp_) versus breakdown voltage characteristics have been calculated for Ga_2_O_3_ Schottky diode devices including the incomplete ionization effect, with the results plotted in Fig. 4. Details of the calculation can be found in the supplementary information. As Fig. 4 shows, the on-resistance versus breakdown voltage characteristics are degraded where the maximum breakdown voltage decreases and the on-resistance increases as the concentration of the 110 meV donor increases. To determine the maximum acceptable concentration of 110 meV donors for a particular target breakdown voltage, the percent increase in R_onsp_ is calculated for a Schottky diode containing both 110 meV donors and shallow donors versus a Schottky diode containing only shallow donors designed for the same breakdown voltage. The results of the calculation are plotted in Fig. 5 with additional details available in the supplementary information. With Fig. 5, we can estimate the maximum concentration of the 110 meV donors acceptable for 10 kV operation of Ga_2_O_3_ Schottky diode devices. To limit the increase in R_onsp_ to one percent, the concentration of 110 meV donors must be less than 5 × 10^14^ cm^−3^. A similar analysis of the percent decrease in breakdown voltage due to 110 meV donors is also presented in the supplementary information.

In conclusion, high temperature Hall effect and admittance spectroscopy measurements have revealed a previously unobserved unintentional donor with energy 110 meV below the conduction band edge in commercially available unintentionally doped Ga_2_O_3_ substrates grown by the EFG method. The existence of these unintentional donors sets a limit on the maximum breakdown voltages that can be achieved in Ga_2_O_3_ devices and must be mitigated to achieve the full benefits of Ga_2_O_3_ based power electronics. Additionally, incomplete ionization of the 110 meV donor causes increased on-resistance and decreased breakdown voltage in diodes containing the donor, as compared to diodes containing only the shallow donor, beyond the usual tradeoff between on-resistance and breakdown voltage. To achieve 10 kV operation in Ga_2_O_3_ Schottky diode devices, analysis indicates that the concentration of 110 meV donors much be reduced below 5 × 10^14^ cm^−3^ to limit the increase in R_onsp_ to one percent.

## Methods

### Sample Fabrication and Measurement

Two van der Pauw test samples were diced from the same two inch wafer into 1 cm × 1 cm square pieces. Following dicing, samples were solvent cleaned and 50 nm/1000 nm Ti/Au contacts were sputtered on the sample corners. To improve contact resistance, the samples were annealed from room temperature up to 450 °C with a 15 min ramp in a tube furnace with argon gas flow. A third sample from the same two inch wafer was prepared for admittance spectroscopy by depositing an indium tin oxide (ITO) transparent contact to form a Ga_2_O_3_ Schottky diode. Following device fabrication, temperature dependent van der Pauw and Hall effect measurements were carried out in two separate Hall effect measurement systems. An electromagnet with vacuum cryostat and closed-loop He refrigerator was used for measurements below room temperature, while an electromagnet with a quartz tube furnace with silicon carbide heater was used for measurements above room temperature. The samples were kept under nitrogen gas flow at atmospheric pressure during high temperature measurements. Admittance spectroscopy measurements were carried out under vacuum in a closed-loop He cryostat using a Keysight Agilent 4990 A impedance analyzer.

### Data Availability

The data that support the findings of this study are available on request from the corresponding author S.M.

## Electronic supplementary material

Supplementary Information
