# Supplementary material for: Incomplete Ionization of a 110 meV Unintentional Donor in β-Ga2O3 and its Effect on Power Devices
Source: Sci Rep. 2017 Oct 16;7:13218. doi: 10.1038/s41598-017-13656-x (PMC5643349; doi:10.1038/s41598-017-13656-x)
Supplement: Supplementary file 1 — Supplementary Information [file 41598_2017_13656_MOESM1_ESM.pdf]

Supplementary Information

Incomplete Ionization of a 110 meV Unintentional Donor in β-Ga_2O_3 and its Effect on Power Devices

Adam T. Neal[1, 2, *], Shin Mou[1, *], Roberto Lopez[3], Jian V. Li[3], Darren B. Thomson[4], Kelson D. Chabak[4], and Gregg H. Jessen[4]

[1] Air Force Research Laboratory, Materials and Manufacturing Directorate, Wright Patterson AFB, OH
[2] Universal Technology Corporation, Dayton, OH
[3] Texas State University, Department of Physics, San Marco, TX
[4] Air Force Research Laboratory, Sensors Directorate, Wright Patterson AFB, OH
[*] Electronic Address: shin.mou.1@us.af.mil and adam.neal.2.ctr@us.af.mil

Temperature Dependent Conductivity and Hall Mobility

The temperature dependence of the Hall mobility is well described by a combination of ionized impurity scattering and polar optical phonon scattering, with a measured mobility of about 430 cm^2/Vs at 140K, 160 cm^2/Vs at room temperature, and 20 cm^2/Vs at 1000K. Fitting of Hall mobility vs temperature yields a maximum room temperature mobility of 230 cm^2/Vs for Ga_2O_3, limited by polar optical phonon scattering, if impurity scattering is sufficiently reduced.

Figure S1 shows the conductivity for the same two samples for which Hall effect measurements are reported in the main text. Figure S2 show the Hall mobility, calculated from the conductivity in Figure S1 below and Hall carrier density from Figure 2 of the main text. The room temperature mobility is measured to be 160 cm^2/Vs, increasing to 430 cm^2/Vs at 140K. As temperature is increased, the mobility decreases to 20 cm^2/Vs. Because Ga_2O_3 is a compound semiconductor, polar optical phonon scattering is expected to dominate at high temperatures while impurity scattering is expected to dominate at low temperatures. Indeed, the temperature dependent mobility is well fit by the combination of these scattering mechanisms, in agreement with the recently published results of Ref 1. The momentum relaxation rates for screened

ionized impurity scattering and polar optical phonon scattering, are as follows.[2] For screened ionized impurity scattering:

$$\frac{1}{\tau_{II}} = \frac{N_I q^4}{16\sqrt{2m^*}\pi\kappa_S^2\varepsilon_0^2}\left[\ln(1+\gamma^2) - \frac{\gamma^2}{1+\gamma^2}\right]E^{-3/2} \tag{S1}$$

$$\gamma^2 = \frac{8m^* E L_D^2}{\hbar^2} \tag{S2}$$

where N_I is the number of ionized impurities and L_D is the Debye screening length. For polar optical phonon scattering:

$$\frac{1}{\tau_{POP}} = \frac{q^2\omega_o\left(\frac{\kappa_S}{\kappa_\infty}-1\right)}{4\pi\kappa_S\varepsilon_0\hbar\sqrt{2[E/m^*]}}\left[N_o\sqrt{1+\frac{\hbar\omega_o}{E}} + (N_o+1)\sqrt{1-\frac{\hbar\omega_o}{E}} - \frac{\hbar\omega_o N_o}{E}\sinh^{-1}\left(\frac{E}{\hbar\omega_o}\right)^{1/2} + \frac{\hbar\omega_o(N_o+1)}{E}\sinh^{-1}\left(\frac{E}{\hbar\omega_o}-1\right)^{1/2}\right] \tag{S3}$$

$$N_o = \frac{M}{e^{\hbar\omega_o/kT}-1} \tag{S4}$$

where N_o is a semi-empirical distribution function for optical phonons with M the effective number of optical phonon modes and $\hbar\omega_o$ the effective optical phonon energy. The total momentum relaxation rate is

$$\frac{1}{\tau_m} = \frac{1}{\tau_{II}} + \frac{1}{\tau_{POP}} \tag{S5}$$

From the solution of the Boltzmann transport equation in the relaxation time approximation for an energy dependent relaxation time, assuming Maxwell-Boltzmann carrier statistics, the mobility is[2]

$$\mu = \frac{q\langle\langle\tau_m\rangle\rangle}{m^*} \tag{S6}$$

$$\langle\langle\tau_m\rangle\rangle = \frac{\int_0^\infty E^{3/2}\tau_m(E)f(E)\,dE}{\int_0^\infty E^{3/2}f(E)\,dE} \tag{S7}$$

and $\langle\langle\tau_m\rangle\rangle$ is a weighted average of the momentum relaxation time over energy specific to transport calculations. The carrier distribution function, $f(E)$, it taken to be the equilibrium distribution function for the mobility calculation performed here. M, $\hbar\omega_o$, and N_I are taken as free parameters to fit the experimental data, while others are estimated from the literature. Table S1 gives a summary of all parameters, and the mobility calculated using Equations S6 and S7 is shown as a solid black line in Figure S2. Individual components of the mobility associated with the two scattering mechanisms are also shown. The resulting fit indicates that ionized impurity scattering and polar optical phonon scattering appropriately describe the temperature dependent mobility of Ga_2O_3. Based on the fitting of polar optical phonon scattering in the Ga_2O_3 samples, it is estimated that the room temperature mobility could be increased from 160 cm^2/Vs to a maximum of 230 cm^2/Vs if the impurity concentration in the sample is further reduced. This fact is illustrated by the calculated polar optical phonon mobility plotted as the dashed line in Figure S2.

Additional Details on the R_{onsp} versus Breakdown Voltage Calculation

To calculate the R_{onsp} versus breakdown voltage characteristics of Figure 4 in the main text, ionized donor concentrations were calculated as a function of the total donor concentrations at room temperature using the charge neutrality equation

$$N_c e^{\frac{E_f - E_c}{kT}} + N_a = \frac{N_{d1}}{1+2e^{\frac{E_f - E_{d1}}{kT}}} + \frac{N_{d2}}{1+2e^{\frac{E_f - E_{d2}}{kT}}} \quad \text{(S8)}$$

with $N_a = 0$ and donor energies as specified in Table I of the main text. R_{onsp} and breakdown voltage were calculated according to the following equations assuming the full-depletion approximation:

$$V_B = \frac{\kappa\, \varepsilon\, E_c^2}{2\, q\, (N_{d1}+N_{d2})} \quad \text{(S9)}$$

$$R_{onsp} = \frac{\kappa\, \varepsilon\, E_c}{\mu\, q^2\, (N_{d1}+N_{d2})\, (N_{d1}^{+}+N_{d2}^{+})} \quad \text{(S10)}$$

The estimated breakdown field of 8 MV/cm for Ga_2O_3 was used for E_c. A room temperature polar optical phonon limited mobility of 230 cm^2/Vs was used for μ, determined from our analysis of Ga_2O_3 scattering mechanisms above. Figure 4 of the main text shows R_{onsp} versus breakdown voltage characteristics generated from Equations S9 and S10 by holding the 110 meV donor concentration N_{d1} fixed while varying the shallow donor concentration N_{d2}.

Additional Details on the Percent Increase in R_{onsp} versus Breakdown Voltage Calculation due to 110 meV donors. Similar Calculation of Percent Decrease in Breakdown Voltage vs. R_{onsp} due to 110 meV donors.

To examine the change in performance of a Schottky diode when 110meV donors are present, the following procedure is adopted and used to generate Figure 5 of the main text and Figure S3 below. To characterize the change in performance, it is useful to compare the performance of a first Schottky diode device with both 110 meV donors and shallow donors to a second baseline Schottky diode device with only shallow donors. Furthermore, as device designers would typically adjust the donor concentration of the drift-layer to target a particular performance metric, breakdown voltage or on-resistance, it makes sense to compare two devices designed to have the same performance metric. Using this approach, we therefore compare a first Schottky diode with a particular concentration of 110 meV donors and a particular concentration of shallow donors to a second baseline Schottky diode with only shallow donors, where the concentration of shallow donors in the second baseline diode is adjusted to match either the breakdown voltage or on-resistance of the first diode. The results of those calculations are shown in Figure 5 of the main text and Figure S3 below. The x-axis of these figures indicates the performance metric, breakdown voltage or on-resistance, which is the same for the two diodes. The y-axis shows the percent change in the other performance metric comparing the two diodes. The calculations were performed for a series of 110 meV donor concentrations N_{d1} ranging from 1×10^{14} cm^{-3} to 1×10^{18} cm^{-3} as labeled in the figures. Again, the shallow donor concentration N_{d2} is adjusted for both diodes to match the particular breakdown voltage or R_{onsp} shown on the x-axis. The symbols and line segments shown in Figures 4 and 5 of the main text and Figure S3 below graphically indicate the relationship between the three figures. The pink

circles and vertical line segment in Figure 4 of the main text illustrate an example increase in R_{onsp} corresponding to the pink circle plotted in Figure 5 of the main text. The cyan squares and horizontal line segment in Figure 4 of the main text illustrate an example decrease in breakdown voltage corresponding to the cyan square plotted in Figure S3 below. The symbols and line segments merely illustrate the relationship of Figure 4 to Figure 5 and Figure S3. The specific values chosen for the illustration are unimportant. With Figure 5 and Figure S3, we can estimate the maximum concentration of the 110 meV donors acceptable for 10 kV operation of Ga_2O_3 Schottky diode devices. Looking at 10 kV in Figure 5, the concentration of 110 meV donors must be less than 5×10^{14} cm^{-3} to limit the increase in R_{onsp} to one percent. Similarly, considering the 10 kV R_{onsp} of 4 $m\Omega.cm^2$ in Figure S3, the concentration of 110 meV donors must be less than 1×10^{15} cm^{-3} to limit the decrease in breakdown voltage to one percent. Of course, this analysis assumes that the shallow donor density can also be sufficiently reduced to achieve 10 kV operation.

Supplementary Information References

1. Ma, N. et al., Intrinsic electron mobility limits in β-Ga_2O_3. *App. Phys. Lett.* **109,** 212101 (2016).
2. Lundstrom, M., *Fundamentals of Carrier Transport, 2nd ed.* Cambridge University Press, 70,86,136-137 (2009)
3. He, H. et al., First-principles study of the structural, electronic, and optical properties of Ga_2O_3 in its monoclinic and hexagonal phases. *Phys. Rev. B* **74,** 195123 (2006).
4. Peelaers, H. and Van, C.G., Brillouin zone and band structure of β-Ga_2O_3. *Phys. Status Solidi (b)* **252,** 828-832 (2015).
5. Furthmüller, J. and Bechstedt, F., Quasiparticle bands and spectra of Ga_2O_3 polymorphs. *Phys. Rev. B* **93,** 115204 (2016).
6. Hoeneisen, B., Mead, C.A., and Nicolet, M.A., Permittivity of β-Ga_2O_3 at low frequencies. *Solid-State Electron.* **14,** 1057-1059 (1971).
7. Schubert, M. et al., Anisotropy, phonon modes, and free charge carrier parameters in monoclinic β-gallium oxide single crystals. *Phys. Rev. B* **93,** 125209 (2016).

8. Rebien, M., Henrion, W., Hong, M., Mannaerts, J.P., and Fleischer, M., Optical properties of gallium oxide thin films. *App. Phys. Lett.* **81,** 250-252 (2002).

9. Sturm, C., Furthmuller, J., Bechstedt, F., Schmidt-Grund, R., and Grundmann, M., Dielectric tensor of monoclinic Ga_2O_3 single crystals in the spectral range 0.5-8.5 eV. *APL Materials* **3,** 106106 (2015).

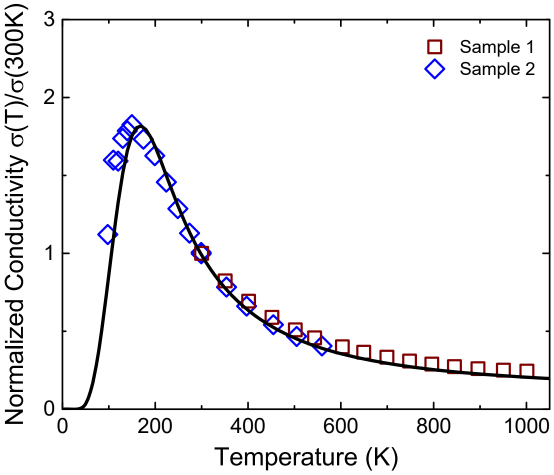

Figure S1: Conductivity vs. temperature of Ga_2O_3 for two samples measured by the van der Pauw method. The data are normalized to the conductivity at 300K. The symbols are the measured data and the black line a fit to the data. Room temperature conductivities are 3.75 S/cm for sample 1 (red square) and 3.11 S/cm for sample 2 (blue diamond).

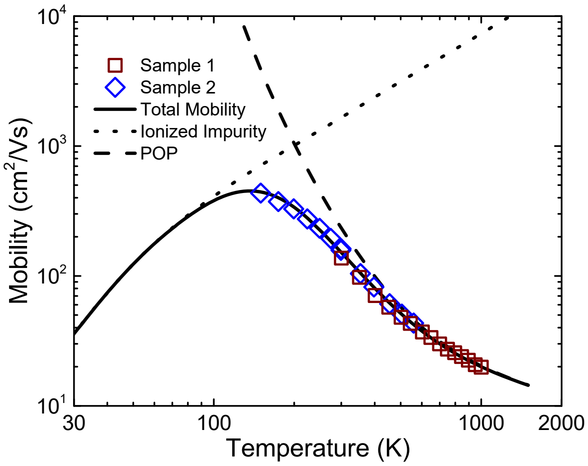

Figure S2: Log-Log plot of Hall mobility vs. temperature Ga_2O_3 for two samples determined from van der Pauw conductivity and Hall effect measurements. The symbols are the measured data and the black line a fit to the data. The two components of the mobility, screened ionized impurity scattering and polar optical phonon scattering, are plotted as dotted and dashed lines, respectively.

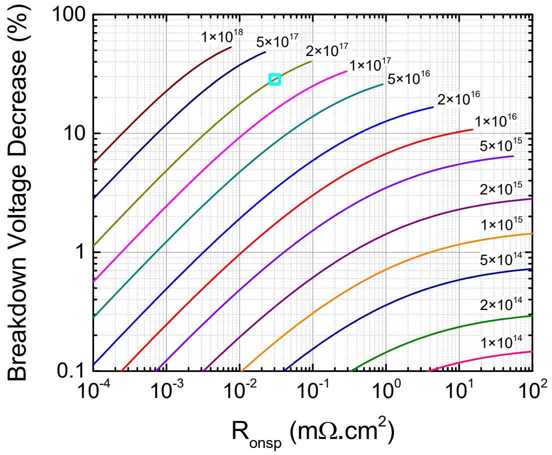

Figure S3: Percent decrease in breakdown voltage due to incomplete ionization as a function of specific on-resistance (R_{onsp}) comparing Ga_2O_3 based Schottky diode devices with both 110 meV donors and silicon donors to devices with only silicon donors. The labels indicate the fixed concentration of 110 meV donors in cm^{-3} for each curve. Note that the percent decrease is calculated for Schottky diodes designed to have the same R_{onsp}, not silicon donor concentration. The cyan square is an example percent decrease in breakdown voltage which corresponds to the cyan squares and horizontal line segment in Figure 4 of the main text. The plotted symbol merely illustrates the relationship to Figure 4. The specific value chosen for the illustration is unimportant.

Table S1: Parameters for the mobility vs. temperature model

[a] N_I	(cm^{-3})	1.8×10^{17}
[a] $\hbar\omega_o$	(meV)	60.
[a] M		1.7
[b] m^*/m_0		0.3
[c] κ_s		10
[d] κ_∞		3.5

[a] free parameters, [b] Ref. 3,4,5 [c] Ref. 6,7
[d] Ref. 7,8,9
